# Molecular Insights into the Mode of Action of Antibacterial Peptides Derived from Chicken Plasma Hydrolysates

**DOI:** 10.3390/foods11223564

**Published:** 2022-11-09

**Authors:** Fu Tian, Sureelak Rodtong, Kanjana Thumanu, Yanling Hua, Sittiruk Roytrakul, Jirawat Yongsawatdigul

**Affiliations:** 1 School of Food Technology, Institute of Agricultural Technology, Suranaree University of Technology, Nakhon Ratchasima 30000, Thailand; 2 School of Pre-Clinics, Institute of Science, Suranaree University of Technology, Nakhon Ratchasima 30000, Thailand; 3 Synchrotron Light Research Institute (Public Organization), Nakhon Ratchasima 30000, Thailand; 4 Center for Scientific and Technological Equipment, Suranaree University of Technology, Nakhon Ratchasima 30000, Thailand; 5 National Center for Genetic Engineering and Biotechnology, Pathumthani 12120, Thailand

**Keywords:** antibacterial peptides, SR-FTIR, molecular docking, *Bacillus cereus*

## Abstract

Due to the overuse and abuse of antibiotics, several antibiotic resistant bacteria have emerged. Antimicrobial peptides (AMPs) have gained attention as alternative antimicrobial agents because of their unique mode of action that impedes bacterial resistance. Two novel antibacterial peptides were isolated from Alcalase-hydrolyzed chicken plasma by size exclusion and reverse-phase chromatography. They were identified by LC-MS/MS to be VSDH and CCCPKAF, which showed effective antibacterial activity toward *Bacillus cereus* DMST 5040, with varied modes of action. The peptide CCCPKAF caused cell membrane disintegration, as evidenced by propidium iodide (PI) uptake. In contrast, the peptide VSDH targeted intracellular molecules, including proteins and nucleic acids, as revealed by Synchrotron-based Fourier Transform Infrared (SR-FTIR). The secondary structure of intracellular proteins increased to a β-sheet structure concomitant with a decrease in the α-helix structure when exposed to 0.5 mM VSDH. Molecular docking analysis revealed that VSDH showed high binding affinity for the active sites of the various enzymes involved in DNA synthesis. In addition, it showed good affinity for a chaperone protein (Dnak), resulting in the misfolding of intracellular proteins. Nuclear magnetic resonance (NMR) and molecular dynamics simulations also indicated that VSDH chelated well with Mg^2+^, which could partly contribute to its antibacterial activity.

## 1. Introduction

Multiple drug-resistant bacteria and the potential health risk of synthetic food preservatives, including allergic reactions, hyperactivity, and cancer, have led to a demand for new strategies for microbial control [1]. Due to the single targets of traditional antibiotics, bacteria may develop tolerances through mutations and changes to antibiotic target sites [2]. Antimicrobial peptides (AMPs) have recently attracted much attention as they act on multiple targets on membrane and intracellular components, such as DNA, enzymes, or proteins [3]. Thus, mutations conferring AMP resistance are less likely to occur.

AMPs can be endogenously found in bacteria, insects, plants, and animals. Utilization of natural AMPs in food and/or pharmaceutical applications is limited by lower yields of extraction and fractionation, except for bacteriocins from bacteria [4]. The approach consisting of protein hydrolysis is more feasible than that of extracting endogenous AMPs. The release of peptides upon hydrolysis by suitable protease(s) could result in peptides that function as AMPs. Pellegrini (1999) reported that the hydrolysis of bovine α-lactalbumin by trypsin and chymotrypsin yielded three peptides with antibacterial activity toward Gram-positive bacteria, but the peptides were less effective against Gram-negative bacteria [5]. The approach consisting of protein hydrolysis is more feasible than that of extracting endogenous AMPs.

Most AMPs reported thus far are cationic peptides containing 10–50 amino acid residues [6]. Positively charged peptides readily bind with negatively charged cell membranes via electrostatic interactions, and the lipid region of the membrane is penetrated and disorganized by the hydrophobic residues of AMPs, causing destabilization of the cell membrane [3]. For this reason, the number of positive charges on peptides are used as an important indicator to screen potential AMPs. However, a few reports have indicated that bacteria could introduce lysine into the cell membrane through MprF synthases to reduce the electronegativity of the cell membrane. Therefore, the probability of AMP binding to the cell membrane is reduced, along with its antimicrobial activity [7]. The new features of cationic AMPs containing fewer positive charges or even anionic peptides should be explored.

The existing analytical tools for elucidating the mechanisms of AMPs include microscopy, flow cytometry, and NMR [8]. The specific targets of AMPs cannot be clearly determined based on these techniques. Fourier transform infrared spectroscopy (FTIR) is an analytical technique that can detect the functional groups of compounds. The main components of bacterial cells have conspicuous characteristic absorption peaks in the infrared region (4000–400 cm^−1^), such as peaks for lipids (3000–2800 cm^−1^), proteins (1700–1500 cm^−1^), and nucleic acids (1300–1100 cm^−1^) [9]. Traditional FTIR with a globar light source presents some limitations, particularly in terms of the diffraction effect of small areas of interest [10]. Alternatively, synchrotron-based Fourier transform infrared (SR-FTIR) can provide a high signal-to-noise ratio (S/N) at ultraspatial resolutions to investigate molecular chemistry within the microstructures of samples. This technique has been widely used to investigate cellular changes in proteins, nucleic acids, and phospholipid components [11,12]. Although FTIR can locate the cellular target components of AMPs, information on the specific target(s) responsible for inhibition is still missing. Molecular docking is another supporting tool that can reveal the plausible individual compounds that interact with AMPs, which could also generate detailed information of the interactions. The use of SR-FTIR in conjunction with molecular docking can provide useful insights into the potential cellular compounds that are targets of the AMPs of interest.

Chicken meat is one of the most important sources of food protein, and approximately 100 million metric tons of chicken meat have so far been produced in 2022 worldwide [13]. Chicken blood of approximately 2.4 to 3.3% of live weight is generated in slaughterhouses [14]. Blood is a good source of nutrients, and it is considered a nonallergenic protein compared with soy and dairy proteins [15]. However, its consumption as food is limited in some cultures, thus it is discarded, causing environmental problems [16,17]. Blood plasma is rich in proteins, including albumin, globulin, and fibronectin. Albumin and globulin have been reported to possess antibacterial properties [18]. Discovering antimicrobial activity in chicken plasma hydrolysate would lead to the valorization of chicken blood.

The objective of this study was to investigate the antibacterial activities of peptides fractionated from chicken blood plasma hydrolysates. In addition, the mode of action of isolated peptides was systematically explored based on bacterial membrane integrity, cell damage, and intracellular reactions. SR-FTIR and molecular docking were applied to gain more insights into the intracellular targets of antibacterial peptides. Finally, NMR and molecular dynamics simulations were applied to determine the metal chelating ability of the identified plasma AMP.

## 2. Materials and Methods

### 2.1. Chicken Plasma Preparation

Fresh chicken blood was collected from a commercial slaughterhouse (Betagro, Lopburi, Thailand) with sodium citrate added to contain a final concentration of 4% to prevent clotting. Upon arrival, the samples were centrifuged at 2000× *g* at 4 °C for 10 min to separate the plasma and blood cells. The plasma fraction was dialyzed using a 3-kDa membrane against deionized water at 4 °C for 18 h. The dialyzed sample was lyophilized, stored at −20 °C, and used throughout the study.

### 2.2. Preparation of Chicken Plasma Hydrolysates

The dialyzed plasma powder was dissolved in deionized water (100 mg/mL) and preincubated at 65.5 °C for 10 min in a water bath. The pH of the plasma solution was adjusted to 9.6, and Alcalase 2.4 L (Novozymes, Bagsvaerd, Denmark) was added at a concentration of 4% (*w*/*w*) of the substrate. The pH was continuously adjusted to 9.6 using 1 M NaOH to maintain the optimal pH for the enzyme during the 6 h hydrolysis reaction. The reaction was terminated by heating the solution at 95 °C for 10 min. Subsequently, the hydrolysates were rapidly cooled to 4 °C in an ice bath, and the pH was adjusted to 7.0 ± 0.1. Supernatants were collected by centrifuging at 10,000× *g* for 20 min and referred to as the chicken plasma hydrolysate (CPH), and they were kept in a −80 °C freezer throughout the study. The degree of hydrolysis of CPH samples was determined using the trinitrobenzenesulfonic acid (TNBS) method [19].

### 2.3. Isolation and Identification of Antibacterial Peptides

One hundred microliters of the diluted CPH was loaded onto a Superdex peptide 10/300 GL column (GE Healthcare Bioscience Co., Uppsala, Sweden), equipped with fast protein liquid chromatography (FPLC; ÄKTA Pure 25, GE Healthcare Bioscience Co., Uppsala, Sweden). The mobile phase was DI water (A) and 30% ACN containing 0.1% trifluoroacetic acid (TFA) (*v*/*v*) (B). Stepwise elution at 0.8 mL/min was performed as follows: 20 mL, 2.5% B; 20 mL, 7.5% B; 20 mL, 12.5% B; 15 mL, 100% B; 35 mL, 2.5% B. The peptides in fractions after gel filtration were detected at 214 nm and 280 nm. Each fraction was collected and lyophilized. The inhibitory effects of these fractions were then tested against *Escherichia coli* and *Staphylococcus aureus*, which were selected as a representative of Gram negative and Gram positive bacteria, respectively. The fraction with the strongest antibacterial inhibitory activity was further purified using a SOURCETM 5RPC ST 4.6/150 column (GE Healthcare Bioscience Co., Uppsala, Sweden). Peptides were eluted by mobile phase A (0.1% TFA in DI water, *v*/*v*) and mobile phase B (0.1% TFA in ACN, *v*/*v*). Stepwise elution at 0.2 mL/min was performed as follows: 5 mL, 2% B; 5 mL, 10% B; 5 mL, 20% B; 5 mL, 30% B; 5 mL, 40% B; and 5 mL, 100% B, adjusted to 2% B for 10 mL. The peptide fractions obtained from RPC were detected and tested as described above.

The fractionated peptide samples were injected into an Ultimate3000 Nano/Capillary LC System (Thermo Scientific, Altrincham, UK) coupled to a Hybrid quadrupole Q-Tof impact II^TM^ (Bruker Daltonics, Billerica, MA, USA) equipped with a nanocaptive spray ion source. The peptides were enriched on a µ-Precolumn (300 µm i.d. × 5 mm, C18 Pepmap 100; Thermo Scientific, Loughborough, UK) and separated with an Acclaim Pepmap RSLC C18 column (2 µm, 100 Å, nanoViper, 75 µm i.d. × 15 cm; Thermo Scientific, Loughborough, UK). Mobile phases A and B containing 0.1% formic acid in water and 0.1% formic acid in 80% acetonitrile, respectively, were applied. A gradient of 5–55% solvent B was used at a constant flow rate of 0.3 μL/min for 30 min. Electrospray ionization was performed using a captive spray at 1.6 kV. MS/MS spectra were obtained in positive-ion mode over the range (*m*/*z*) 150–1000 (Compass 1.9 software, Bruker Daltonics, Billerica, MA, USA), and data were analyzed using PEAKS Studio 10.0 software (Waterloo, ON, Canada). Only peptides with “de novo” scores above 70% were selected for further analysis.

### 2.4. Antibacterial Assay

The antibacterial activity was determined using a microbroth activity assay [20]. Briefly, *S*. *aureus* ATCC 25923, *B. cereus* DMST 5040, *E*. *coli* ATCC 25992, and *Salmonella* Typhimurium TISTR 292 were cultured at 37 °C in trypticase soy broth (TSB), and pure colonies were obtained from trypticase soy agar (TSA). *Listeria monocytogenes* D17303 were cultured at 37 °C in TSB containing 0.6% yeast extract, and pure colonies were obtained from TSA containing 0.6% yeast extract. A single colony was diluted in Mueller–Hinton broth (MHB) to 1 × 10^5^ CFU/mL. *S*. *aureus*, and *E*. *coli* were used as target bacteria for CPH and peptide fractions from chromatographic separation. Identified peptides that were chemically synthesized were tested for their antibacterial activity against 5 bacterial strains. Briefly, fifty microliters of bacterial cultures was added to microtiter plate wells containing 50 μL of peptide samples and incubated at 37 °C for 18 h. Turbidity was measured at 600 nm (Varioskan LUX Multimode Microplate Reader, Thermo Fisher Scientific, Waltham, MA, USA). Kanamycin at 20 ppm and DI water were prepared as positive and negative controls, respectively. The minimum inhibitory concentration (MIC) of the identified peptides was determined. Twofold serial dilutions of each sample were incubated with each culture in a 96-well microplate at 37 °C for 18 h. The lowest concentration of the peptide that showed growth inhibition as evaluated by the absorbance at 600 nm was determined to be the MIC.

### 2.5. Integrity of Cell Membranes

#### 2.5.1. Leakage of Nucleotides

DNA leakage was analyzed by measuring the absorbance at 260 nm according to Gao et al. [21], with some modifications. *B. cereus* in 10 mM phosphate buffered saline (PBS) was adjusted to 1 × 10^6^ CFU/mL. Then, 2 × MIC of each synthetic peptide was added at a ratio of 1:1 (*v*/*v*) and incubated at 37 °C. After time intervals of 0, 2, 4, and 6 h, the cell cultures were centrifuged at 3000 rpm, and the absorbance of the supernatant was measured at 260 nm using a μDropTM Plate A 2-3 (Thermo Scientific, Waltham, MA, USA) and a Varioskan LUX Multimode microplate reader (Thermo Fisher Scientific, Waltham, MA, USA). PBS at 10 mM was used as the negative control.

#### 2.5.2. Cell Membrane Disintegration

Cell membrane integrity was detected by confocal laser scanning microscopy (CLSM) following Wang et al. [22], with some modifications. Briefly, *B. cereus* cells (3 × 10^8^ CFU/mL) in the mid-logarithmic phase were incubated with synthetic peptides at 1 × MIC for 1 h. The cells were centrifuged at 1500× *g* for 5 min, washed 3 times with 10 mM PBS, and resuspended with propidium iodide (PI) at a final concentration of 10 μg/mL in 10 mM PBS (pH 7.2). After incubation for 30 min at 4 °C, the unbound dye was removed by washing with excess PBS. Control cells were prepared in the absence of peptides. Then, 10 μL of the cell suspension was transferred onto a glass slide and observed using a Nikon A1Rsi CLSM at an excitation wavelength of 535 nm. Localization of the peptide targets followed Wang et al. [22], with some modifications. *B*. *cereus* cells (3 × 10^8^ CFU/mL) were incubated with an FITC-labeled peptide at 1×MIC for 1 h. The sample was then centrifuged at 1000× *g* for 10 min, washed 3 times with 10 mM PBS, and resuspended with PI (10 μg/mL) in PBS. After incubation for 30 min at 4 °C, the unbound PI dye was removed by washing with excess PBS. The samples were smeared onto a glass slide and observed using a Nikon A1Rsi CLSM at excitation wavelengths of 488 and 535 nm for FITC and PI, respectively.

### 2.6. Morphological Characteristics

The effect of synthetic peptides on the morphology of *B. cereus* was investigated according to Vishwesharaiah et al. [23], with some modification. Mid-logarithmic cells were treated with peptides at 1 × MIC for 1 and 4 h. Subsequently, the cells were harvested and fixed with 2.5% glutaraldehyde at 4 °C overnight and dehydrated with 20% to 100% acetone. The dried cells were coated with carbon. The specimens were observed at an accelerating voltage of 3 kV and at a magnification of 10,000 by scanning electron microscopy (SEM) (ZEISS Gemini, Carl Zeiss, Jena, Germany).

### 2.7. Intracellular Changes

#### 2.7.1. Changes of Biomolecules by SR-FTIR

Bacterial cells were prepared following the method of Naksang et al. [24], with some modifications. *B*. *cereus* cells were grown at 37 °C in TSB with the addition of synthetic peptides at a final concentration of 1 × MIC. The cells were taken at 0 and 4 h and centrifuged at 3000× *g* for 10 min. The cell pellets were washed 3 times with sterile 0.85% NaCl and DI water. The suspended culture was placed on a BaF_2_ window, dried under vacuum for 2 h, and stored in a desiccator to form a film before analysis. SR-FTIR microspectroscopy was carried out with a Beamline 4.1 instrument at the Synchrotron Light Research Institute (SLRI, Nakhon Ratchasima, Thailand). The FTIR spectra were obtained in a range of 800–3000 cm^−1^. All spectra were smoothed, normalized, and baseline corrected, and the averages of the spectra were calculated using an OPUS 7.5 (Bruker, Ettlingen, Germany). Significant variations among data groups were identified by principal component analysis (PCA) in the spectral range of 3000–2800 and 1800–900 cm^−1^ using an Unscrambler 10.4 software (CAMO, Oslo, Norway). Data were processed by taking the second derivative using the Savitzky–Golay algorithm, with seventeen points of smoothing to minimize the effects of variable baselines. The processed data were normalized with extended multiplicative signal correction (EMSC) to account for differences in sample thickness, correcting for scattering artifacts.

#### 2.7.2. Interactions of DNA with VSDH

Interaction of DNA with VSDH was investigated using gel retardation assay. Briefly, the DNA extraction of *B. cereus* was carried out according to Neumann et al. [25]. The extracted DNA (700 ng) was mixed with 0.5, 1 or 2 mM of VSDH Tris-HCl buffer (10 mM, pH = 8) in 10 μL and incubated at 37 °C for 1 h. The ability of the peptides to bind with DNA was analyzed using agarose gel electrophoresis (1%). Gel retardation was visualized under UV illumination using a gel imaging system (Bio–Rad, Hercules, CA, USA).

### 2.8. Molecular Docking Studies

#### 2.8.1. Construction of the Homology Model

Thymidylate kinase (TMK), thymidylate synthase (TS), dihydrofolate reductase (DHFR), and DNA gyrase subunits A and B were selected as targets for molecular docking because they are related to DNA synthesis. In addition, a chaperone protein (DnaK) was also selected because it is related to protein folding. Because the crystallographic structures of these proteins of *B*. *cereus* were not available, comparative modes were constructed using the I-TASSER server [26]. The sequence for each protein was obtained from the UniProt database. The I-TASSER server generated five separate models from the FASTA input, and the top-ranked model was selected based on the best available C-score. The C-score is the estimated global accuracy of the model, denoted as a value between [−5, 2], with a score greater than −1.5 indicating a model of correct global topology [27].

#### 2.8.2. Docking

AutoDock Vina was used to predict binding conformations and affinities between the peptide (VSDH) and each enzyme. Docking simulations were performed according to Trott & Olson [28]. The structure of the VSDH was generated by PyMol 2.5. A grid box with dimensions of 50 Å × 50 Å × 50 Å was made to cover the entire binding pocket, including the active site with a specific grid spacing of 0.375 Å. The 3D structure picture file was prepared by PyMol 2.5. Vina scores were given as the predicted affinity of the VSDH to bind to each enzyme, which was calculated in kcal/mol.

### 2.9. Metal Chelating Ability of VSDH

#### 2.9.1. Effect of Metal Ions on the Growth of *B*. *cereus*

*B. cereus* was preincubated with VSDH (1 × MIC) at 37 °C for 18 h in MH broth. Turbidity was measured at 600 nm. Subsequently, 0.25 mM and 0.5 mM Mg^2+^ were added separately. The samples were further incubated for another 10 h, and turbidity was measured at 600 nm.

#### 2.9.2. Mg^2+^-Binding Ability of VSDH

The Mg^2+^-binding ability of VSDH was studied by nuclear magnetic resonance (NMR). Briefly, VSDH (20 mM) and MgCl_2_ (20 mM) were mixed in DI water at 37 °C for 30 min. The mixture was analyzed on a 500 MHz NMR spectrometer (Bruker AVANE III HD) with a CCP BBO 500 Cyroprobe at 25 °C. Deuterated water was used as the solvent. ^1^H and ^13^C NMR spectra were collected at frequencies of 500.363 and 125.816, respectively. The Bruker NMR software Topspin 3.5pl6 was used for data collection and data processing.

#### 2.9.3. Stability of Mg^2+^-VSDH Complex by Molecular Dynamics (MD) Simulations

To verify the stability of binding, MD simulations were used. Briefly, the MD simulations were performed on a Yinfo Cloud Computing Platform (https://cloud.yinfotek.com (accessed on 5 Novermber 2021)). The 3D structure of the peptide was generated by the platform tool and then used to manually construct the initial model of the peptide–metal complex. MD simulations were executed using an AmberTools20 package [29], with the AMBER ff19SB [30] force filed. The system was solvated by a cubic water box using an OPC water model with a margin of 12 Å. Periodic boundary conditions (PBCs) were applied, and the net charge was neutralized by Na^+^ (or Cl^−^). To remove improper atom contacts, the structure was first minimized by 2500 steps of steepest descent and 2500 steps of conjugate gradient, under a harmonic constraint of 10.0 kcal/(mol·Å2) on heavy atoms, then by 2500 steps of steepest descent and 2500 steps of conjugate gradient, under the same harmonic constraint on protein backbone atoms, and finally by 10,000 steps of steepest descent and 10,000 steps of conjugate gradient without constraint. After being fully minimized, the system was gradually heated to 300 K by a 20 ps NVT simulation. Subsequently, two-step equilibration phases were carried out: (1) a 200 ps NPT simulation with constraints on heavy atoms followed by (2) a 1000 ps NVT simulation without constraints. The temperature was maintained at 300 K using the Berendsen thermostat with a 1 ps coupling constant, and the pressure was maintained at 1 atm using a Monte Carlo barostat with a 1 ps relaxation time. Finally, the system was subjected to a 20 ns NVT simulation. The root-mean-square deviation (RMSD) was analyzed by the CPPTRAJ module.

### 2.10. Statistical Analysis

Statistical analysis was performed using the GraphPad Prism software version 9, and statistical significance was determined using one-way ANOVA followed by Tukey’s multiple comparison test when more than 2 groups were compared. A *t*-test was used when only 2 groups were compared. Significance levels are presented by the number of asterisks: * *p* < 0.05, ** *p* < 0.01, *** *p* < 0.001, and **** *p* < 0.0001.

## 3. Results and Discussion

### 3.1. Effect of Plasma Hydrolysates on Bacterial Growth

**Figure 1:**
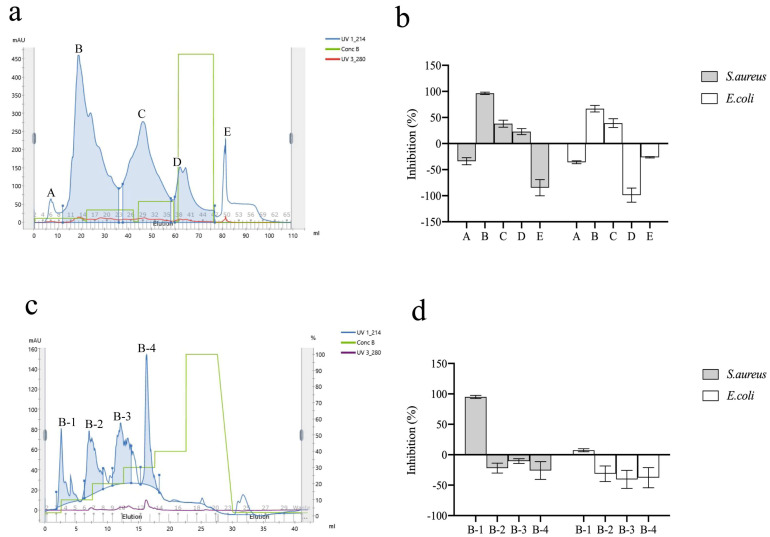
(**a**) Size exclusion chromatography of peptides from CPH (A-E are peptide peaks ); (**b**) The antibacterial activities of different peptide fractions obtained by size−exclusion chromatography (4 mM *L*−Leu equivalents); (**c**) RPC of peptides from Peak B; (**d**) The antibacterial activities of different peptide fractions obtained by RPC (2 mM *L*−Leu equivalents). Data are presented as the mean ± SD, *n* = 3.

CPH with a degree of hydrolysis of 27.7% did not inhibit the growth of *E*. *coli* and *S*. *aureus*, which agreed with previous studies on the plasma hydrolysates of cattle, sheep, deer, and pigs [20,31]. After size-exclusion chromatography, five fractions were obtained, with peak B having the most effective antibacterial activity, with approximately 94% and 69% inhibition toward *S*. *aureus* and *E*. *coli*, respectively (Figure 1a,b). Peak D showed different trends, as it inhibited *S*. *aureus* but promoted the growth of *E*. *coli*. The plasma hydrolysates likely contained both nutrient peptides and antimicrobial peptides against *S*. *aureus* and *E*. *coli*. Based on these results, peak B was selected for further purification using an RPC column.

Four fractions were obtained after RPC (Figure 1c). Only peak B-1 showed antibacterial ability toward *S*. *aureus*, with 96.44% growth inhibition (Figure 1d). Inhibition of *E*. *coli* was not found after RPC chromatography. Although multistep purification can result in higher purity, some active substances are lost [32]. The yield of peptides from RPC was estimated to be 1.9%. Based on these results, Peak B-1 was selected for peptide identification.

### 3.2. Identification of Antibacterial Peptides

A total of seven peptides were identified in peak B-1, and their characteristics are summarized in Table S1. The MS/MS spectra of these peptides are displayed in Figure S1. All identified peptides showed positively charged and hydrophobic residues, which have been reported to play a role in antibacterial activity [33]. These peptides were considered novel peptides based on the antibacterial peptide database (http://aps.unmc.edu (accessed on 10 May 2020)). Among the seven peptides tested with five microorganisms, VSDH inhibited both *S*. *aureus* and *B*. *cereus*, while CCCPKAF only showed antibacterial activity toward *B*. *cereus* (Figure S2). The MIC values of VSDH and CCCPKAF against *B. cereus* were 0.5 mM and 0.25 mM, respectively. In addition, the MIC of VSDH against *S. aureus* was 0.5 mM. Both peptides showed weak inhibition towards *S. typhimirium* and *L*. *monocytogenes* with MIC > 8 mM. They did not inhibit *E*. *coli*. The MIC value of VSDH against *S*. *aureus* was lower than that of peptide Mop2 (HVLDTPLL) from *Moringa oleifera* seed protein hydrolysate at 2.2 mM [34] and SP-1 (KLVDASHRLATGDVAVRA) from the protein hydrolysate of *Spirulina platensis* at 8.52 mM [35]. The peptide AVDRAV, from laba garlic, showed a more effective MIC value of 0.1 mM [21]. As VSDH and CCCPKAF shared common inhibition toward *B*. *cereus*, the mode of action of these two peptides as anti-bacillus agents was further studied.

Most antibacterial peptides are amphiphilic and positively charged with net charges of +2 to +9. Peptides with a greater number of positive charges can promote better interaction with the cell membrane, resulting in membrane disruption [3]. The peptides (VSDH and CCCPKAF) identified in our study contained K and R, with net charges of 0 and +1, respectively, and showed good antibacterial ability against *B. cereus*. The smaller and more amphiphilic peptides can diffuse through the membrane to exert greater bacteriostatic effects [22]. This might explain why the peptide VSDH, with a relatively smaller mass, exhibited good inhibitory activity. For the peptide CCCPKAF, the positive charge (+1) may contribute to the interaction with the negatively charged bacterial membrane. In addition, the cysteine (C) and proline (P) in CCCPKAF might contribute to its stronger antibacterial activity. An important role of the C residue is to form disulfide bonds to stabilize the β-hairpin or sheet structure [36]. In addition, the P residue is conducive to the formation of a linear structural conformation for AMPs and increases membrane permeability [37]. Our peptides did not show antimicrobial activity against Gram-negative bacteria. AMPs specific for Gram-negative bacteria must adsorb onto the lipopolysaccharide on the outer membranes and penetrate through the cytoplasmic membrane to induce membrane disintegration. Such peptides typically contain both positive charges and hydrophobic characteristics [38]. Our results suggested that peptides with small molecular masses and fewer positively charged residues also exhibited antimicrobial properties and might possess different antimicrobial mechanisms from those of classic AMPs.

### 3.3. Mechanisms of Antibacterial Activity

#### 3.3.1. Leakage of Nucleotides

When the bacterial membrane is damaged, DNA or RNA can be released from cells, which can be detected at 260 mm. The cells treated with CCCPKAF showed a drastic increase in absorbance during the first 2 h of exposure and remained stable afterward (Figure S3). However, VSDH did not induce any leakage of nucleotides. As a shorter peptide, VSDH was unlikely to cause cell membrane damage.

#### 3.3.2. Morphological Characteristics

**Figure 2:**
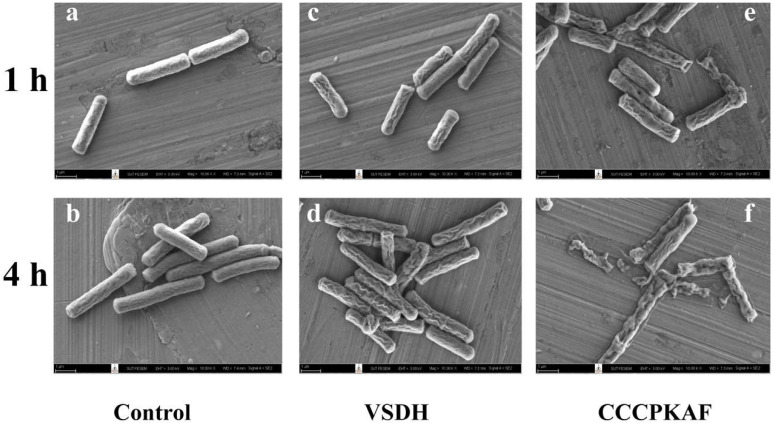
Morphological changes of *B. cereus* treated with 0.5 mM VSDH and 0.25 mM CCCPKAF equivalent to 1 × MIC, observed by scanning electron microscopy: Control 1 and 4 h (**a**,**b**); VSDH 1 and 4 h (**c**,**d**); CCCPKAF 1 and 4 h (**e**,**f**).

In the control group (without peptide treatment), *B. cereus* showed typical cell morphological characteristics with regular rod shapes and smooth surfaces (Figure 2a,b). After treatment with VSDH for 1 h and 4 h, deformations and wrinkles on the cellular surface were clearly observed (Figure 2c,d). It should be noted that holes were not observed. These results suggest that VSDH penetrated the bacterial cell membranes without severely compromising their integrity. Miao et al. [39] identified a dipeptide containing leucine and tyrosine connected with two phosphate molecules from kefir, which inhibited the growth of *E*. *coli* and *S*. *aureus* without causing severe cell membrane damage. When treated with CCCPKAF for 1 h, *B*. *cereus* cells exhibited significant alterations, with noticeable holes and deformed cell structures (Figure 2e). Prolonged exposure over 4 h resulted in cell collapse (Figure 2f). These cell abnormalities indicated that CCCPKAF might have caused disintegration of the cell membranes, leading to cell death. Gao et al. [21] also observed that incubation of *E*. *coli* and *S*. *aureus* with peptides isolated from laba garlic caused morphological changes. These results indicated that VSDH and CCCPKAF affected cell membranes in varied fashion.

#### 3.3.3. Cell Membrane Disintegration

**Figure 3:**
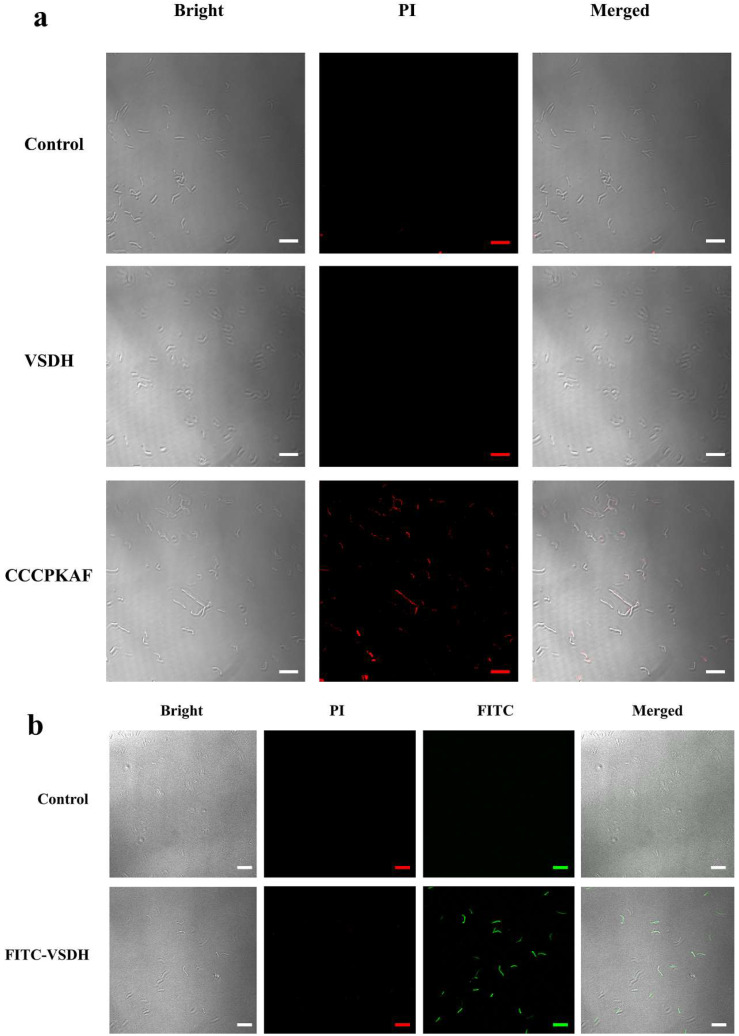
CLSM images of *B. cereus* treated with chicken blood plasma peptides: (**a**) *B*. *cereus* treated with VSDH and CCCPKAF and PI, the red signal from PI. (**b**) *B*. *cereus* treated with FITC-labeled VSDH and PI, green signal from FITC peptides.

To investigate the membrane-perturbing activity of VSDH and CCCPKAF, CLSM was applied (Figure 3a). PI is a membrane impermeable dye that stains nucleic acids in cells when the cell membrane is destroyed [22]. The control (cells without peptide) displayed no appreciable fluorescent signal, indicating limited PI uptake. In contrast, cells emitted red fluorescence after treatment with 0.25 mM CCCPKAF (1 × MIC) for 1 h, confirming that CCCPKAF destroyed the integrity of the cell membrane. However, cells treated with 0.5 mM VSDH (1 × MIC) for 1 h did not show an obvious red fluorescent signal, suggesting that the cell membrane integrity was intact after 1 h of exposure. This result indicated that VSDH penetrated cells and caused a bactericidal effect. The FITC-labeled VSDH also demonstrated that VSDH was localized in the cytoplasm without a distinct PI fluorescent signal (Figure 3b). These results confirmed that VSDH was absorbed intracellularly without disturbing the integrity of the cell membranes.

#### 3.3.4. Changes of Biomolecules by SR-FTIR

**Figure 4:**
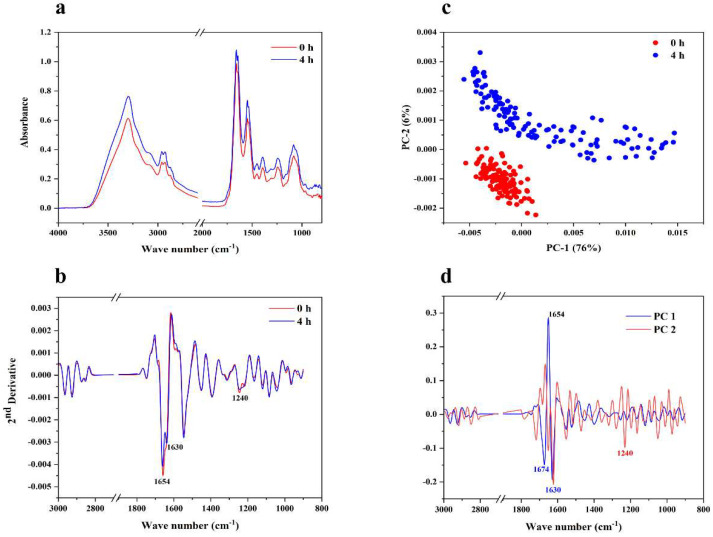
SR−FTIR analysis of *B. cereus* treated with VSDH at 4 h. (**a**) Representative average IR absorbance; (**b**) Second derivatives of FTIR spectra; (**c**) PCA scores plot; (**d**) their corresponding loadings plots.

The effect of VSDH on the intracellular components of *B*. *cereus* was further investigated using SR-FTIR microspectroscopy. The FTIR spectra for cells can be divided into three regions, namely, cellular fatty acids (3000–2800 cm^−1^), the amide groups of proteins and peptides (1700–1500 cm^−1^), and the phosphate group asymmetric stretching of nucleic acids (1350–1000 cm^−1^) [9]. After VSDH exposure for up to 4 h, changes in the intracellular components of *B*. *cereus* were evident (Figure 4a). In Figure 4b, peak shifts were noticed in the amide I (1654 cm^−1^ and 1630 cm^−1^), α-helix (1654 cm^−1^), and β-sheet (1630 cm^−1^) structures [40]. Among them, the peak at 1630 cm^−1^ was slightly higher after 4 h of treatment, indicating the unfolding of intracellular proteins to more β-sheet structures. A peak shift was also observed at 1240 cm^−1^, representing the phosphate group (P=O) asymmetric stretching of the phosphodiester bond of nucleic acids. These changes suggest that VSDH is likely an intracellular protein and a nucleic acid of *B*. *cereus*.

The PCA score plot of SR-FTIR demonstrated a clear separation between samples exposed to the peptide for 0 and 4 h (Figure 4c). According to a loading plot (Figure 4d), the separation was found to be associated with amide I (1674, 1650, and 1630 cm^−1^) and nucleic acids (1240 cm^−1^). The results from SR-FTIR spectroscopy agreed with those of CLSM and SEM, which indicated that the VSDH passed through the membrane without significantly disturbing its integrity. VSDH mainly altered intracellular proteins and nucleotides, which might be one of the major causes of its antimicrobial activity.

#### 3.3.5. Interactions of DNA with VSDH

Based on SR-FTIR, notable changes in nucleic acids were observed by VSDH treatment. Based on the in vitro tests, VSDH did not cause any changes in DNA migration at concentrations up to 2 mM, indicating a lack of peptide-DNA binding. The peptide VSDH contains a negatively charged amino acid (D), which hampers binding to negatively charged DNA. Thus, VSDH might interfere with DNA synthesis rather than exerting an effect through direct binding.

### 3.4. Molecular Docking

**Figure 5:**
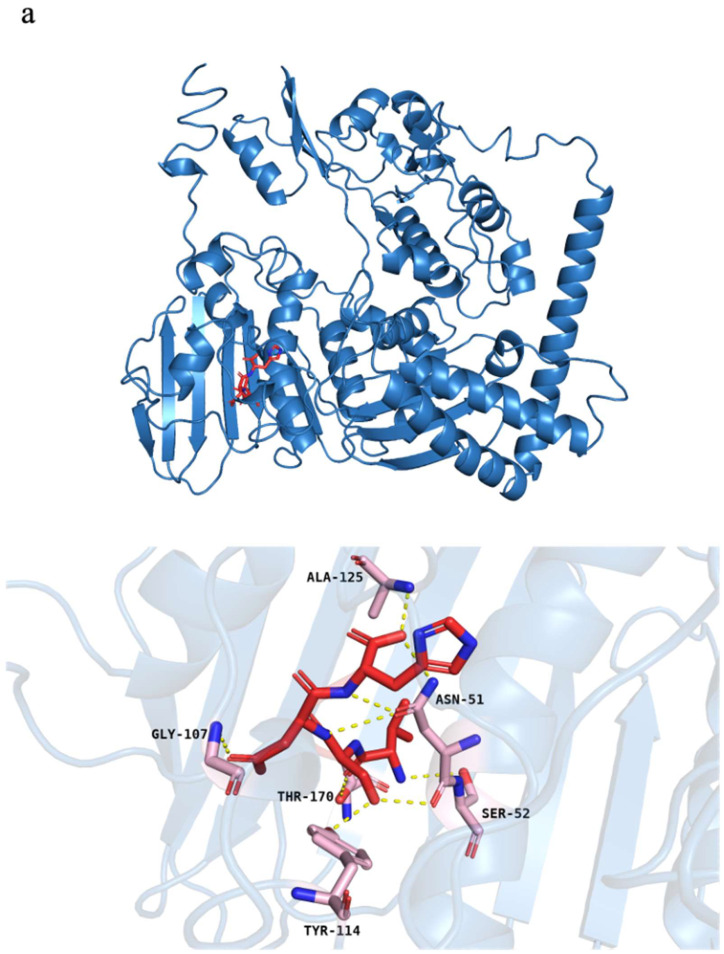

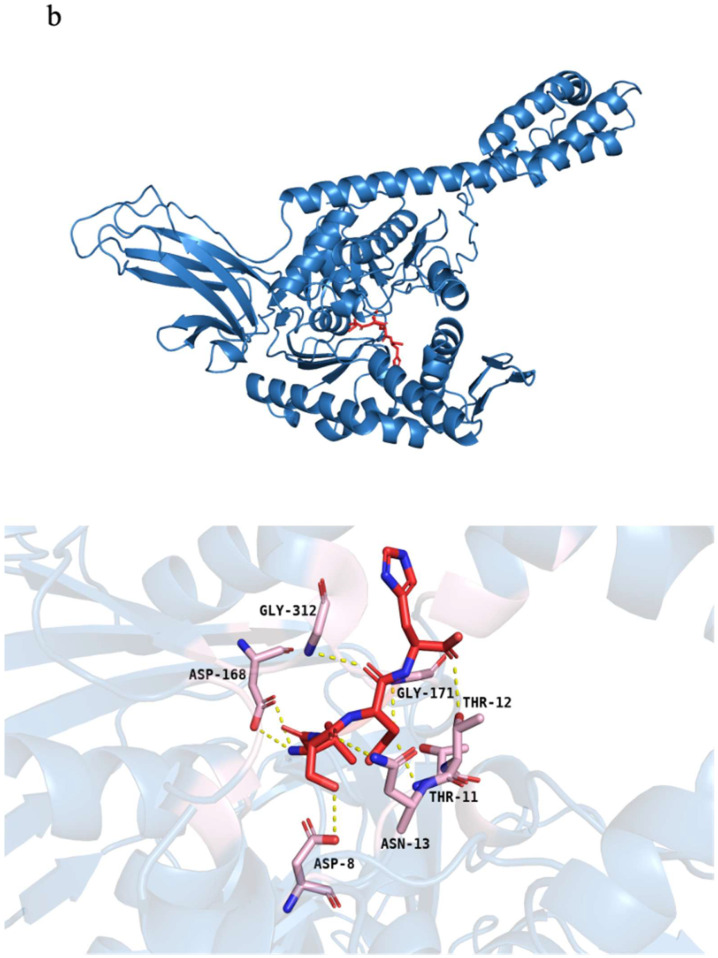
Molecular docking of VSDH with enzymes related to DNA synthesis. (**a**) DNA gyrase subunit B; (**b**) DnaK. The backbone of the VSDH peptide is depicted in red lines; the active sites of both enzymes are depicted in pink lines; the yellow dots indicate hydrogen bonds.

To gain more insights into how DNA interferes with VSDH, molecular docking was applied to elucidate interactions between the peptide and essential enzymes related to DNA synthesis, namely, thymidylate kinase (TMK), thymidylate synthase (TS), dihydrofolate reductase (DHFR), and DNA gyrase subunit A and subunit B. Among the enzymes tested, the DNA gyrase subunit B showed the greatest peptide binding affinity of −8.4 kcal/mol (Table S2). The binding model suggested that binding between VSDH and the DNA gyrase subunit B occurred via hydrogen bonds involving Gly 107, Thr 170, Tyr 114, Ser 52, Asn 51, and Ala 125 in the binding pocket (Figure 5a). DNA gyrase plays a key role in the process of DNA replication, transcription, and chromosome separation. DNA gyrase helps alleviate torsional tension by introducing negative supercoils to DNA molecules during DNA replication [41]. Thangaraj et al. [42] also reported that quinoline peptides strongly interacted with DNA gyrase, inhibiting the growth of *B. cereus*. In addition, DHFR is an enzyme catalyzing the reduction of dihydrofolate to tetrahydrofolate, thereby promoting thymidylate biosynthesis and improving DNA translation, RNA transcription, and protein replication [43]. Thus, it is vital for controlling cell proliferation. Based on molecular docking, VSDH showed good binding affinity to DHFR (Table S2), which would inhibit the enzyme activity and ultimately limit cell growth. DHFR inhibitors have been proven to be effective agents for treating bacterial infections, including those caused by *Mycobacterium tuberculosis*, *S*. *aureus*, and *E*. *coli* [43]. Our results indicated that DNA gyrase and DHFR could be major targets for VSDH, interfering with DNA and protein synthesis, as evidenced by the SR-FTIR spectra.

Based on the SR-FTIR spectra, the alteration of intracellular proteins was evident after VSDH treatment. Kragol et al. [44] reported that pyrrhocoricin bound to the bacterial heat shock protein DnaK and affected chaperone-assisted protein folding, resulting in the growth inhibition of *E*. *coli*. Thus, the interaction between DanK of *B*. *cereus* and VSDH was elucidated. The docking score for VSDH and DanK was −9.2 kcal/mol, indicating good binding affinity. VSDH formed more hydrogen bonds with amino acid residues, including Asp 168, Asp 8, Asn 13, Thr 12, Thr 11, Gly 171, and Gly 312 (Figure 5b). These results imply that VSDH also induced protein misfolding, rendering changes in protein functions and ultimately inhibiting growth. It can be seen from the docking results that VSDH interfered with both DNA and protein synthesis in *B. cereus*. However, these results should be further confirmed by the transcriptomics and proteomics of *B*. *cereus*.

### 3.5. Metal Chelating Ability of VSDH

**Figure 6:**
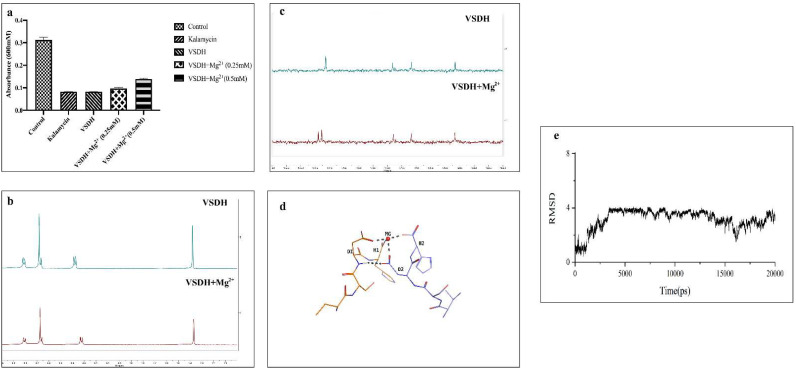
Metal chelating ability of VSDH during the growth of *B. cereus*. (**a**) Cells were treated with 0.5 mM VSDH for 18 h, and two concentrations of Mg^2+^ were added for an additional 10 h. Optical density was determined at 600 nm. Data are presented as the mean ± SD, *n* = 3; (**b**) ^1^H NMR spectra of the interaction between VSDH and Mg^2+^; (**c**) ^13^C NMR spectra of the interaction between VSDH and Mg^2+^; (**d**) 3D structure of the VSDH-Mg^2+^ complex; (**e**) Root mean square deviation (RMSD) plot for the VSDH-Mg^2+^ complex during 20 ns of molecular dynamics simulation.

Histidine-rich AMPs can complex with metal ions via two nitrogen atoms of the imidazole ring [45,46]. Corbin et al. [47] reported that calprotectin, a protein found in human neutrophils, inhibits the growth of several bacteria and fungi by zinc and manganese chelation. Microplusin, an antimicrobial peptide isolated from the Cattle Tick *Rhipicephalus Boophilus microplus*, inhibited *Micrococcus luteus* due to its copper II chelating ability. VSDH has a histidine residue at the C-terminus and contains a negatively charged amino acid (D). Moreover, the amino acid residues of DH resemble those of microplusin, which plays an important role in binding copper II [48]. Magnesium (Mg^2+^) is an essential trace element for protein synthesis that neutralizes the charge on ribosomal RNA to allow the proper folding of proteins. Insufficient intracellular Mg^2+^ ions lead to the disassembly of ribosomes [49]. Mg^2+^ is also vital for DNA synthesis as it is essential for DNA polymerase, catalyzing the incorporation of deoxynucleoside triphosphates into a growing DNA chain [50]. Thus, it was hypothesized that the Mg^2+^ chelating ability of VSDH might play some roles in its antibacterial ability. When *B. cereus* was cultured with VSDH for 18 h, Mg^2+^ (0.25 and 0.5 mM) was added to the medium, and incubation was prolonged for another 10 h. Growth appeared to recover at 0.5 mM Mg^2+^ addition (Figure 6a). Chelation of Mg^2+^ induced the metal starvation of *B*. *cereus*, leading to its limited growth. Growth recovery upon Mg^2+^ addition confirmed the VSDH chelation ability. The 1H NMR spectra also illustrated that the NH group showed a chemical shift at 8.32 ppm in the presence of Mg^2+^ (Figure 6b). The ^13^C NMR (Figure 6c) spectra also revealed coordination between the C=O of Asp or the C-terminus and Mg^2+^, as distinct peaks at approximately 174.2 ppm were observed. The possible chelation between VSDH and Mg^2+^ is illustrated in Figure 6d. The C-termini of the two molecules VSDH and Asp-COOH were coordinated with Mg^2+^. To verify the chelation mechanism, a peptide–metal complex was established, and a 20 ns molecular dynamics simulation was performed (Figure 6d). The root mean square deviation (RMSD) (Figure 6e) during 20 ns implied high binding stability between VSDH and Mg^2+^. Thus, these results demonstrated that VSDH chelated well with Mg^2+^, which could be another vital mode of antimicrobial action.

## 4. Conclusions

The two novel antibacterial peptides, VSDH and CCCPKAF, isolated from chicken plasma hydrolysates displayed unique characteristics with different antibacterial modes of action toward *B*. *cereus.* CCCPKAF exhibited a lower positive charge and disturbed the integrity of the cell membrane, which is a typical characteristic of AMPs. In contrast, VSDH is a short neutral peptide showing multiple mechanisms. It passed through the cell membrane and reacted with various intracellular targets, including proteins and DNA. Molecular docking revealed that VSDH showed good binding affinity to the DNA gyrase subunit B and DnaK, resulting in the inhibition of DNA synthesis and in protein misfolding, respectively. VSDH also chelated Mg^2+^, depriving an essential trace element for growth. These two peptides showed potential as emerging antimicrobial agents for food and pharmaceutical applications.

## Data Availability

Date is contained within the article or Supplementary Material.

## Supplementary Materials

The following supporting information can be downloaded at: https://www.mdpi.com/article/10.3390/foods11223564/s1, Figure S1: MS/MS spectra of seven peptides in peak B-1 identified by LC–MS/MS; Figure S2. Antibacterial activities of various synthetic peptides derived from CPH at 2 mM. Data are presented as the mean ± SD, *n* = 3; **** *p* < 0.0001, n.s.: not significant (*p* > 0.05, compared with kanamycin); Figure S3. Effect of VSDH and CCCPKAF on DNA leakages from *B. cereus*. Data are presented as the mean ± SD, *n* = 3; **** *p* < 0.0001; n.s.: not significant at *p* > 0.05 compared with PBS; Table S1: Characteristics of antibacterial peptides of CPH identified by LC-MS/MS; Table S2. Docking energy of various enzymes related to DNA synthesis.

## References

[1] Anand S.P., Sati N. (2013). Artificial preservatives and their harmful effects: Looking toward nature for safer alternatives. Int. J. Pharm. Sci. Res..

[2] Von Wintersdorff C.J.H., Penders J., van Niekerk J.M., Mills N.D., Majumder S., van Alphen L.B., Savelkoul P.H.M., Wolffs P.F.G. (2016). Dissemination of antimicrobial resistance in microbial ecosystems through horizontal gene transfer. Front. Microbiol..

[3] Brogden K.A. (2005). Antimicrobial peptides: Pore formers or metabolic inhibitors in bacteria?. Nat. Rev. Microbiol..

[4] Tang W., Zhang H., Wang L., Qian H. (2014). Membrane-disruptive property of a novel antimicrobial peptide from anchovy (*engraulis japonicus*) hydrolysate. Int. J. Food. Sci. Technol..

[5] Pellegrini A., Thomas U., Bramaz N., Hunziker P. (1999). Isolation and identication of three bactericidal domains in the bovine k-lactalbumin molecule. Biochim. Biophys. Acta.

[6] Mahlapuu M., Håkansson J., Ringstad L., Björn C. (2016). Antimicrobial peptides: An emerging category of therapeutic agents. Front. Cell. Infect. Microbiol..

[7] Nawrocki K., Crispell E., McBride S. (2014). Antimicrobial peptide resistance mechanisms of gram-positive bacteria. Antibiotics.

[8] Li S., Wang Y., Xue Z., Jia Y., Li R., He C., Chen H. (2021). The structure-mechanism relationship and mode of actions of antimicrobial peptides: A review. Trends Food Sci. Technol..

[9] Wang M., Lu X., Yin X., Tong Y., Peng W., Wu L., Li H., Yang Y., Gu J., Xiao T. (2015). Synchrotron radiation-based fourier-transform infrared spectromicroscopy for characterization of the protein/peptide distribution in single microspheres. Acta Pharm. Sin. B.

[10] Yu P. (2004). Application of advanced synchrotron radiation-based fourier transform infrared (sr-ftir) microspectroscopy to animal nutrition and feed science: A novel approach. Br. J. Nutr..

[11] Clède S., Lambert F., Sandt C., Gueroui Z., Delsuc N., Dumas P., Vessières A., Policar C. (2013). Synchrotron radiation ftir detection of a metal-carbonyl tamoxifen analog. correlation with luminescence microscopy to study its subcellular distribution. Biotechnol. Adv..

[12] Jamin N., Miller L., Moncuit J., Fridman W.-H., Dumas P., Teillaud J.-L. (2003). Chemical heterogeneity in cell death: Combined synchrotron ir and fluorescence microscopy studies of single apoptotic and necrotic cells. Biopolymers.

[13] Chicken Meat Production Worldwide from 2012 to 2022. https://www.statista.com/statistics/237637/production-of-poultry-meat-worldwide-since-1990/.

[14] Hamzeh A., Wongngam W., Kiatsongchai R., Yongsawatdigul J. (2019). Cellular and chemical antioxidant activities of chicken blood hydrolysates as affected by in vitro gastrointestinal digestion. Poult. Sci..

[15] Sorapukdee S., Narunatsopanon S. (2017). Comparative study on compositions and functional properties of porcine, chicken and duck blood. Korean J. Food Sci. Anim. Resour..

[16] Wongngam W., Mitani T., Katayama S., Nakamura S., Yongsawatdigul J. (2020). Production and characterization of chicken blood hydrolysate with antihypertensive properties. Poult. Sci..

[17] Peña-Saldarriaga L.M., Fernández-López J., Pérez-Alvarez J.A. (2020). Quality of chicken fat by-products: Lipid profile and colour properties. Foods.

[18] Arzumanyan V.G., Ozhovan I.M., Svitich O.A. (2019). Antimicrobial effect of albumin on bacteria and yeast cells. Bull. Exp. Biol. Med..

[19] Adler-Nissen J. (1979). Determination of the degree of hydrolysis of food protein hydrolysates by trinitrobenzenesulfonic acid. J. Agric. Food Chem..

[20] Bah C.S.F., Bekhit A.E.-D.A., Carne A., McConnell M.A. (2015). Production of bioactive peptide hydrolysates from deer, sheep and pig plasma using plant and fungal protease preparations. Food Chem..

[21] Gao X., Chen Y., Chen Z., Xue Z., Jia Y., Guo Q., Ma Q., Zhang M., Chen H. (2019). Identification and antimicrobial activity evaluation of three peptides from laba garlic and the related mechanism. Food Funct..

[22] Wang J., Song J., Yang Z., He S., Yang Y., Feng X., Dou X., Shan A. (2019). Antimicrobial peptides with high proteolytic resistance for combating gram-negative bacteria. J. Med. Chem..

[23] Vishweshwaraiah Y.L., Acharya A., Hegde V., Prakash B. (2021). Rational design of hyperstable antibacterial peptides for food preservation. NPJ Sci. Food.

[24] Naksang P., Tongchitpakdee S., Thumanu K., Oruna-Concha M.J., Niranjan K., Rachtanapun C. (2020). Assessment of antimicrobial activity, mode of action and volatile compounds of etlingera pavieana essential oil. Molecules.

[25] Neumann B., Pospiech A., Schairer H.U. (1992). Rapid isolation of genomic dna from gram-negative bacteria. Trends Genet..

[26] Roy A., Kucukural A., Zhang Y. (2010). I-TASSER: A unified platform for automated protein structure and function prediction. Nat. Protoc..

[27] Yang J., Zhang Y. (2015). I-TASSER server: New development for protein structure and function predictions. Nucleic Acids Res..

[28] Trott O., Olson A.J. (2010). AutoDock Vina: Improving the speed and accuracy of docking with a new scoring function, efficient optimization, and multithreading. J. Comput. Chem..

[29] Case D.A., Francisco S., Belfon K., Ben-Shalom I.Y., Brozell S.R., Cerutti D.S., Cheatham T.E., Cruzeiro V.W.D., Darden T.A., Duke R.E. (2020). Amber 2020.

[30] Tian C., Kasavajhala K., Belfon K.A.A., Raguette L., Huang H., Migues A.N., Bickel J., Wang Y., Pincay J., Wu Q. (2020). Ff19SB: Amino-acid-specific protein backbone parameters trained against quantum mechanics energy surfaces in solution. J. Chem. Theory Comput..

[31] Bah C.S.F., Carne A., McConnell M.A., Mros S., Bekhit A.E.-D.A. (2016). Production of bioactive peptide hydrolysates from deer, sheep, pig and cattle red blood cell fractions using plant and fungal protease preparations. Food Chem..

[32] Tang W., Zhang H., Wang L., Qian H. (2014). New cationic antimicrobial peptide screened from boiled-dried anchovies by immobilized bacterial membrane liposome chromatography. J. Agric. Food Chem..

[33] Dashper S.G., Liu S.W., Reynolds E.C. (2007). Antimicrobial peptides and their potential as oral therapeutic agents. Int. J. Pept. Res. Ther..

[34] Zhao Q., He L., Wang X., Ding X., Li L., Tian Y., Huang A. (2022). Characterization of a novel antimicrobial peptide isolated from *moringa oleifera* seed protein hydrolysates and its membrane damaging effects on *Staphylococcus aureus*. J. Agric. Food Chem..

[35] Sun Y., Chang R., Li Q., Li B. (2016). Isolation and characterization of an antibacterial peptide from protein hydrolysates of spirulina platensis. Eur. Food. Res. Technol..

[36] Bastos P., Trindade F., da Costa J., Ferreira R., Vitorino R. (2018). Human antimicrobial peptides in bodily fluids: Current knowledge and therapeutic perspectives in the postantibiotic era. Med. Res. Rev..

[37] Graf M., Mardirossian M., Nguyen F., Seefeldt A.C., Guichard G., Scocchi M., Innis C.A., Wilson D.N. (2017). Proline-rich antimicrobial peptides targeting protein synthesis. Nat. Prod. Rep..

[38] Lei J., Sun L., Huang S., Zhu C., Li P., He J., Mackey V., Coy D.H., He Q. (2019). The antimicrobial peptides and their potential clinical applications. Am. J. Transl. Res..

[39] Miao J., Guo H., Chen F., Zhao L., He L., Ou Y., Huang M., Zhang Y., Guo B., Cao Y. (2016). Antibacterial effects of a cell-penetrating peptide isolated from kefir. J. Agric. Food Chem..

[40] Barth A., Zscherp C. (2002). What vibrations tell about proteins. Quart. Rev. Biophys..

[41] Ashley R.E., Dittmore A., McPherson S.A., Turnbough C.L., Neuman K.C., Osheroff N. (2017). Activities of gyrase and topoisomerase iv on positively supercoiled dna. Nucleic Acids Res..

[42] Thangaraj M., Gengan R.M., Ranjan B., Muthusamy R. (2018). Synthesis, molecular docking, antimicrobial, antioxidant and toxicity assessment of quinoline peptides. J. Photochem. Photobiol. B Biol..

[43] He J., Qiao W., An Q., Yang T., Luo Y. (2020). Dihydrofolate reductase inhibitors for use as antimicrobial agents. Eur. J. Med. Chem..

[44] Kragol G., Lovas S., Varadi G., Condie B.A., Hoffmann R., Otvos L. (2001). The antibacterial peptide pyrrhocoricin inhibits the atpase actions of dnak and prevents chaperone-assisted protein folding. Biochemistry.

[45] Brewer D., Lajoie G. (2000). Evaluation of the metal binding properties of the histidine-rich antimicrobial peptides histatin 3 and 5 by electrospray ionization mass spectrometry. Rapid Commun. Mass Spectrom..

[46] Nishikawa M., Ogawa K. (2004). Antimicrobial activity of a chelatable poly(arginyl-histidine) produced by the ergot fungus *Verticillium kibiense*. Antimicrob. Agents Chemother..

[47] Corbin B.D., Seeley E.H., Raab A., Feldmann J., Miller M.R., Torres V.J., Anderson K.L., Dattilo B.M., Dunman P.M., Gerads R. (2008). Metal chelation and inhibition of bacterial growth in tissue abscesses. Science.

[48] Silva F.D., Rezende C.A., Rossi D.C.P., Esteves E., Dyszy F.H., Schreier S., Gueiros-Filho F., Campos C.B., Pires J.R., Daffre S. (2009). Structure and mode of action of microplusin, a copper ii-chelating antimicrobial peptide from the cattle tick rhipicephalus (boophilus) microplus. J. Biol. Chem..

[49] Pontes M.H., Yeom J., Groisman E.A. (2016). Reducing ribosome biosynthesis promotes translation during low mg^2+^ stress. Mol. Cell.

[50] Bermek O., Grindley N.D.F., Joyce C.M. (2011). Distinct roles of the active-site mg^2+^ ligands, asp882 and asp705, of dna polymerase i (klenow fragment) during the prechemistry conformational transitions. J. Biol. Chem..

