# Supplementary material for: Molecular Insights into the Mode of Action of Antibacterial Peptides Derived from Chicken Plasma Hydrolysates
Source: Foods. 2022 Nov 9;11(22):3564. doi: 10.3390/foods11223564 (PMC9689829; doi:10.3390/foods11223564)
Supplement: Supplementary file 1 [file foods-11-03564-s001.zip › foods-1977074-supplementary.pdf]

foods

Supplementary Materials

Molecular Insights into the Mode of Action of Antibacterial Peptides Derived from Chicken Plasma Hydrolysates

Fu Tian [1], Sureelak Rodtong [2], Kanjana Thumanu [3], Yanling Hua [4], Sittruk Roytrakul [5] and Jirawat Yongsawatdigul [1,*]

[1] School of Food Technology, Institute of Agricultural Technology, Suranaree University of Technology, Nakhon Ratchasima 30000, Thailand
[2] School of Pre-Clinics, Institute of Science, Suranaree University of Technology, Nakhon Ratchasima 30000, Thailand
[3] Synchrotron Light Research Institute (Public Organization), Nakhon Ratchasima 30000, Thailand
[4] Center for Scientific and Technological Equipment, Suranaree University of Technology, Nakhon Ratchasima 30000, Thailand
[5] National Center for Genetic Engineering and Biotechnology, Pathumthani 12120, Thailand
* Correspondence: jirawat@sut.ac.th; Tel.: +66-44224359

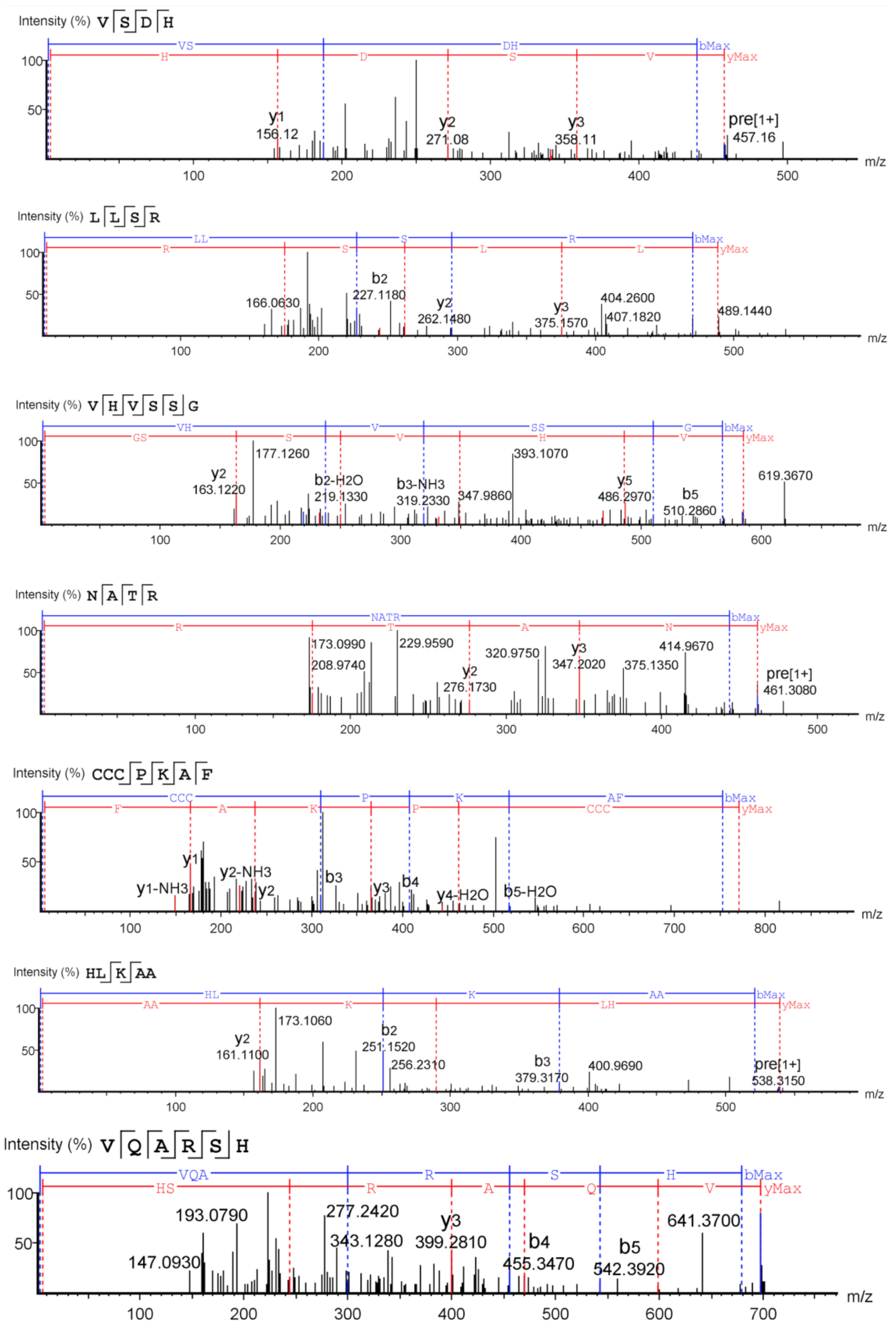

Figure S1. MS/MS spectra of seven peptides in peak B-1 identified by LC–MS/MS.

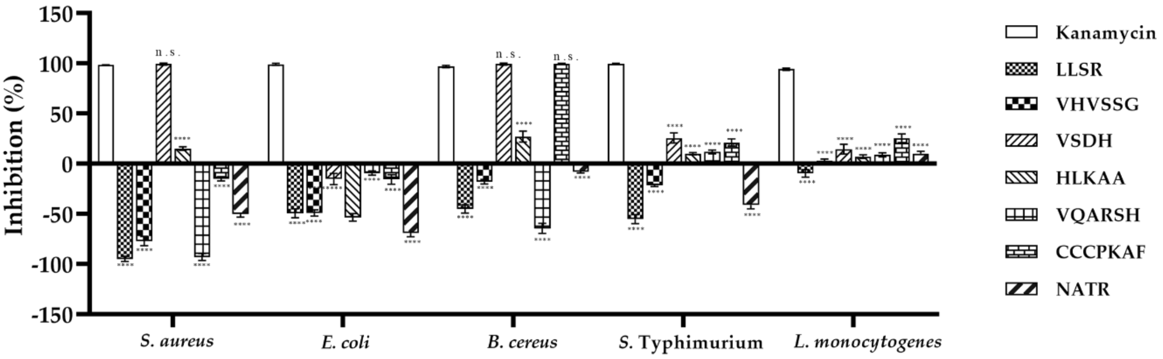

Figure S2. Antibacterial activities of various synthetic peptides derived from CPH at 2 mM. Data are presented as the mean ± SD, n=3; ****p < 0.0001, n.s.: not significant (p > 0.05, compared with kanamycin).

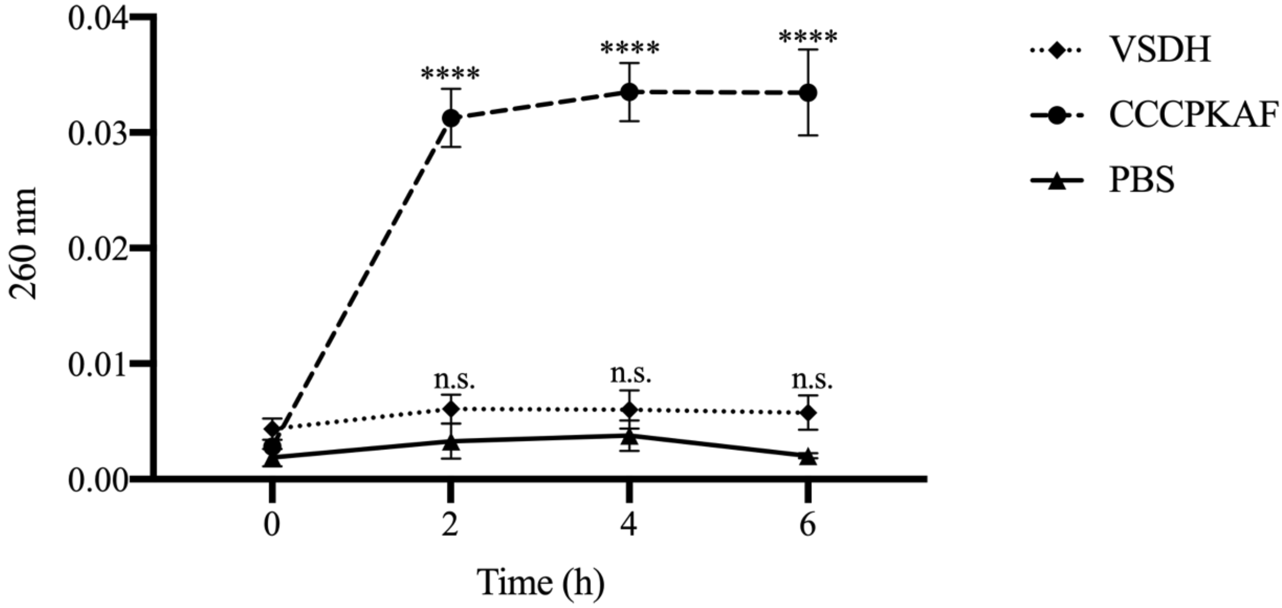

Figure S3. Effect of VSDH and CCCPKAF on DNA leakages from B. cereus. Data are presented as the mean±SD, n=3; ****p < 0.0001; n.s.: not significant at p > 0.05 compared with PBS.

Table S1. Characteristics of antibacterial peptides of CPH identified by LC-MS/MS.

Peptide	MS)Da(	ALC)%(	Net charge	Parent protein
VSDH	457.17	75	0	Protein transport protein Sec31A
LLSR	488.30	84	1	SCO-spondin
VHVSSG	585.33	75	1	SCO-spondin
NATR	461.28	83	1	SCO-spondin
CCCPKAF	771.30	70	1	Immunoglobulin-like domain-containing receptor 2 isoform X1
HLKAA	539.27	75	2	Iron-dependent oxygenase
VQARSH	697.38	70	2	Cyclin-J isoform X1

Table S2. Docking energy of various enzymes related to DNA synthesis.

Receptor	Docking energy)kcal/mol(
DNA gyrase subunit B	-8.4
DHFR	-7.7
TS	-7.1
DNA gyrase subunit A	-6.7
TMK	-6.4
